# Compound Fault Characteristic Analysis for Fault Diagnosis of a Planetary Gear Train

**DOI:** 10.3390/s24030927

**Published:** 2024-01-31

**Authors:** Yulin Ren, Guoyan Li, Xiong Li, Jingbin Zhang, Runjun Liu, Sifan Shi

**Affiliations:** 1 Key Laboratory of Advance Transducers and Intelligent Control System, Ministry of Education, Taiyuan University of Technology, Taiyuan 030024, China; renyulin991003@163.com (Y.R.); 19873375060@163.com (X.L.); zhangjingbin04@163.com (J.Z.); 13277234451@163.com (R.L.); ssf18838282021@163.com (S.S.); 2 School of Mechanical and Electrical Engineering, University of Electronic Science and Technology of China, Chengdu 611731, China

**Keywords:** planetary gear train, carrier eccentricity error, dynamic modeling, compound fault

## Abstract

The carrier eccentricity error and gear compound faults are most likely to occur simultaneously in an actual planetary gear train (PGT). Various faults and errors are coupled with each other to generate a complex dynamic response, which makes the diagnosis of PGT faults difficult in practice. In order to analyze the joint effect of the error and the compound faults in a PGT, a carrier eccentricity error model is proposed and incorporated into the TVMS model by considering the time-varying center distance, line of action (LOA), meshing angle, and contact ratio. Then, the TVMS of the cracked gear is derived based on the potential energy method. On this basis, the dynamic model of a PGT with both the carrier eccentricity error and compound gear cracks as internal excitations are established. Furthermore, the meshing characteristics and dynamic responses of the PGT are simulated to investigate the compound fault features. A series of experiments are conducted to further analyze the influence of the compound fault on the vibration response. The relevant conclusions can provide a reference for the compound fault diagnosis of a PGT in practice.

## 1. Introduction

### 1.1. Literature Review

Planetary gear trains (PGT) are critical for the motion and power transmission of industry applications such as wind turbines, helicopters, construction machinery, etc. The key parts of a PGT are usually subjected to long-term alternating loads due to the time-varying operating conditions. As a result, multiple structural damages will be induced, which has a significant impact on the operational safety of mechanical equipment. In addition, most of the internal components of a PGT work in conjunction with each other; if multiple faults with different degrees simultaneously occur in several key parts, the fault characteristics are often coupled with each other and the performance degradation law of the system is extremely complex. Therefore, the compound fault diagnosis of a PGT becomes an important research direction [1,2].

Considering the compound effect between different faults, it is difficult to extract the compound fault characteristics from the raw signal due to the influence of multi-source external excitation, such as variable operating conditions, strong noise, and multi-interface attenuation. A series of research papers have been produced on compound fault feature extraction. Wang et al. [3] proposed a Resonance Sparse Signal Decomposition (RSSD) and an improved MOMEDA method to decouple the compound fault of the bearing and planet. Lyu et al. [4] improved the Maximum Correlation Kurtosis Deconvolution (MCKD) method to diagnose the compound fault of the bearing and planet. Zhao et al. [5] used the Generalized VD-Kalman Filter (GVKF) to eliminate the influence of speed fluctuation and irrelevant harmonic components. Chen et al. [6] proposed a physics-informed hyperparameter selection strategy for LSTM identification and subsequently the fault detection of gearboxes. He et al. [7] used a sparse representation of vibration signals to effectively diagnose the compound fault of the bearing and gear in a fixed shaft gearbox. Above all, since the compound faults will behave as a complex vibration, most of the research mainly focuses on the decoupling of the compound fault signal. However, it is essential to investigate the correlation between compound fault characteristics and system performance degradation to accurately separate and identify multiple fault features.

Fault dynamic modeling is an effective way to reveal the compound fault mechanism. Moshrefzadeh et al. [8] established a lumped parameter model to study the dynamic response of planet-bearing compound faults. Ma et al. [9] studied the impact of different faults on the dynamic characteristics of gear transmission systems. Wang et al. [10] established dimensionless dynamic equations for a two-stage fixed shaft gear with crack faults and a one-stage planetary gear train with wear faults, then analyzed the bifurcation and spectral characteristics of the system. Ouyang et al. [11] established a dynamic model of a spur-geared rotor system with pitting-crack compound faults and then discussed the evolution process of compound faults and their impact on time-varying meshing stiffness. Chen et al. [12] extracted the crack fault with different depths by means of the fast spectral kurtosis method (FSK) and verified the validity of the Lempel–Ziv index as a damage index for crack depths. Chen et al. [13] used the extended finite element method (XFEM) to simulate the three-dimensional crack propagation path of spur gears under partial load and established the relevant dynamic finite element models and multi-body dynamic models to analyze the dynamic characteristics of the gear-rotor system. However, the abovementioned dynamic models were formed by assuming that the components were ideal and the influence of multi-source errors was not discussed, which is not consistent with the actual situation.

Some researchers have already noticed that manufacturing and assembly errors, such as geometric eccentricity error, pinhole position error, tooth profile error, and so on, can induce complex modulations and dynamic excitations, which will further influence the fault dynamic characteristics [14,15,16,17]. In particular, the carrier eccentricity error is most common and inevitable in an actual PGT. Zhao et al. [18] noticed that the carrier eccentricity error can change the center distance, LOA, meshing angle, and contact ratio of the meshing gear pairs. In addition, they also discovered that the carrier eccentricity error could generate the modulation sidebands around the gear-meshing frequency of normal PGT. However, the above research mainly focuses on the impact of eccentricity errors on the dynamic behavior of normal PGT, such as transmission errors, contact characteristics, and uniform load characteristics. The influence of carrier eccentricity error on the fault dynamic characteristics was not discussed.

Time-varying meshing stiffness (TVMS) modeling is critical for the fault dynamic analysis of gear systems. TVMS considering different fault sets have been derived based on the energy method, the finite element method, the Ishikawa method, and so on. For TVMS with various faults, Mo et al. [19] proposed a TVMS calculation model for a helical gear pair with cracks, and studied the effects of the crack initiation width, termination width, effective contact length, and crack propagation angle on the gear. Yang et al. [20] proposed an improved TVMS calculation method for chipped gear teeth, and the dynamic response of corresponding faults in the gear rotor system was further studied. Doğan et al. [21] introduced the numerical crack propagation paths to the 3D CAD geometries and evaluated the effects of the backup ratio and tooth asymmetry on the spur gears’ meshing stiffness characteristics. For the TVMS of other fault modes, Meng et al. [22] analyzed the influence of different pitting morphologies on TVMS based on a matrix equation. Tian et al. [23] proposed a finite element wear model to explore the three-dimensional tooth surface wear distribution and calculate the corresponding TVMS. Shen et al. [24] proposed a computational model that quantifies the impact of tooth wear on TVMS based on the potential energy method and combined it with the Archard wear equation to calculate the depth of tooth wear. However, almost all of them neglected the influence of eccentricity error on TVMS. In particular, the carrier eccentricity error of PGT will make the actual position of the planet deviate from its ideal positions, which will induce time-varying center distance, LOA, meshing angle, and contact ratio. As a result, the TVMS of the meshing pairs will be directly affected. The dynamic responses can be even more complex for a PGT with both carrier eccentricity error and compound gear faults.

### 1.2. Main Work of This Paper

For an actual PGT, the carrier eccentricity error and multiple faults are most likely to occur simultaneously. However, the compound fault mechanism and the influence of carrier eccentricity error on the fault characteristics are still limited, which leads to more challenges in compound fault diagnosis in practice.

In this paper, a carrier eccentricity error model is proposed, which is then incorporated into the TVMS model by considering the time-varying center distance, LOA, meshing angle, and contact ratio. Next, the TVMS of cracked gear is derived based on the potential energy method. Based on this foundation, the fault dynamic model of a PGT with both carrier eccentricity error and compound gear faults as internal excitations are established. Further, the TVMS characteristics and dynamic responses of a PGT with the carrier eccentricity error and various faults are simulated to investigate the vibration characteristics and coupling mechanisms. Finally, experiments are conducted to further analyze the correctness of the relevant coupling mechanism. The research results can provide a reference for the compound fault diagnosis of a PGT in practice.

### 1.3. Contributions and Innovations

(1) A carrier eccentricity error model is proposed considering the time-varying center distance, LOA, meshing angle, and contact ratio.
(2) An improved TVMS model of a PGT considering both the carrier eccentricity error and compound gear faults is established.
(3) The engagement characteristics and dynamic responses of a PGT are investigated to reveal the vibration responses and coupling mechanisms of compound gear faults and the carrier eccentricity error.

## 2. Improved TVMS Model Considering the Carrier Eccentricity Error and Gear Cracks

### 2.1. Overview of PGT

**Figure 1:**
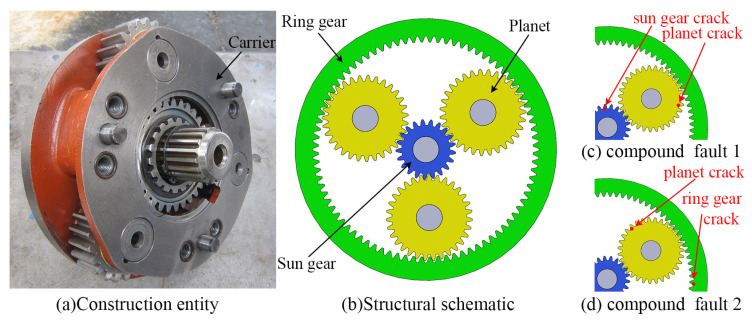
K-H planetary gear train.

The structure of a PGT for construction machinery is shown in Figure 1a,b. The sun gear *s* is set as the input component and the ring gear *r* is kept fixed. The carrier *c* is the output component, on which *N* planets are installed. The compound fault settings in this paper consist of the faults shown in Figure 1c,d. The specification of all equation symbols and nomenclature in this paper is given in Appendix A.

### 2.2. The Carrier Eccentricity Error Model

**Figure 2:**
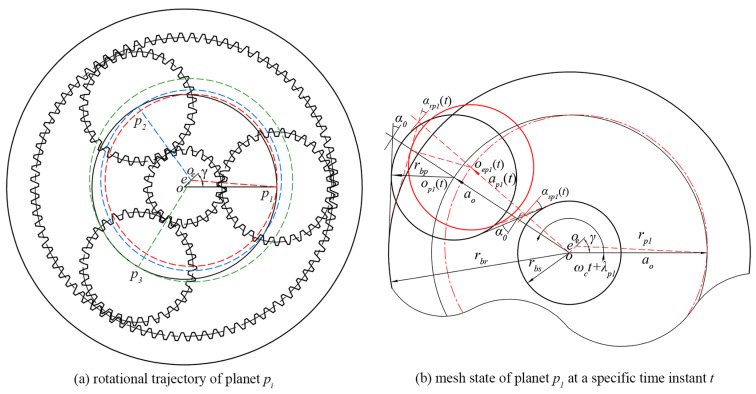
Variations of mesh positions with carrier eccentricity error.

The carrier eccentricity error is inevitable for the actual PGT in the manufacturing and assembling process. As the carrier eccentricity error acts, the PGT operates with the following problems: Each planet cannot operate according to its theoretical trajectory, and the actual trajectory of each planet is shown in Figure 2a, which leads to a time-varying deviation of the meshing center distance. Subsequently, the LOA, meshing angle, and contact ratio will be time-varying as the planetary gear train operates.

In Figure 2a, o and $o_{e}$ are the theoretical and actual rotation centers of the carrier, respectively. The carrier eccentricity error is defined by two parameters, the magnitude *e* and phase angle *γ*. Ideally, each planet would rotate around the center point *o* and form the theoretical trajectory shown by the solid black curve. As the carrier eccentricity error acts, planet $p_{i}$ will rotate around the center $o_{e}$ with radius $r_{pi}$. At this point, the radius of each planet is in a different radius from the others, and together they form the rotational trajectories indicated by the red, blue, and green dotted lines, respectively.

The actual radius $r_{pi}$ is expressed as:

(1) $$r_{pi}=\sqrt{r_{0}{\;}^{2}+e^{2}-2r_{0}e\cos\left(\lambda_{pi}-\gamma \right)}$$

where $r_{0}$ denotes the theoretical radius of planet $p_{i}$. $\lambda_{pi}$ means the initial position angle of planet $p_{i}$ and $\lambda_{pi}=\frac{\left[2\pi \left(i-1\right)\right]}{N}$.

The meshing state of planet $p_{1}$ at a specific time instant t is shown in Figure 2b. $o_{p1}\left(t\right)$ and $o_{ep1}\left(t\right)$ represent the theoretical and actual meshing positions of planet $p_{1}$ respectively. $a_{0}$ is the theoretical center distance. $r_{bj}$ represents the base circle radius of gear *j*.

Based on the actual radius $r_{pi}$ and the meshing relationship, the actual center distance $a_{pi}(t)$ can be expressed as:

(2) $$a_{pi}\left(t\right)=\sqrt{r_{pi}^{2}-e^{2}\left[1-\cos^{2}\left(\omega_{c}t+\pi +\lambda_{pi}-\gamma \right)\right]}-e\cos\left(\omega_{c}t+\pi +\lambda_{pi}-\gamma \right)$$

where $\omega_{c}$ is the carrier rotating speed.

The actual meshing angle can be written as:

(3) $$\alpha_{g}\left(t\right)=\cos^{-1}\left[\frac{m\left(z_{1}+z_{2}\right)\cos\left(\alpha_{0}\right)}{a_{pi}\left(t\right)}\right]$$

where $g=sp_{i},rp_{i}$ represent the sun gear–planet meshing pair and the ring gear–planet meshing pair, respectively. *m* is the gear module. $z_{1}$ and $z_{2}$ are the tooth number of the driving and driven gear, respectively. $\alpha_{0}$ is the theoretical meshing angle.

The actual contact ratio can be expressed as:

(4) $$\varepsilon_{g}\left(t\right)=\frac{1}{2\pi }\left[z_{1}\left(\tan\alpha_{a1}-\tan\alpha_{g}\left(t\right)\right)\pm z_{2}\left(\tan\alpha_{a2}-\tan\alpha_{g}\left(t\right)\right)\right]$$

where $\alpha_{a1}$ and $\alpha_{a2}$ represent the addendum meshing angle of the driving gear and the driven gear, respectively. The symbol + is used for the $sp_{i}$ meshing pair and—for the $rp_{i}$ meshing pair.

### 2.3. Improved TVMS Model Considering the Carrier Eccentricity Error

TVMS is a critical internal excitation of the PGT. The planetary carrier eccentricity error affects the contact ratio and contact angle through the center distance, which will lead to time-varying fluctuations in the engagement interval and amplitude of TVMS, respectively. In this section, an improved TVMS model considering the carrier eccentricity error is derived.

#### 2.3.1. Single-Tooth Meshing Stiffness Derivation

**Figure 3:**
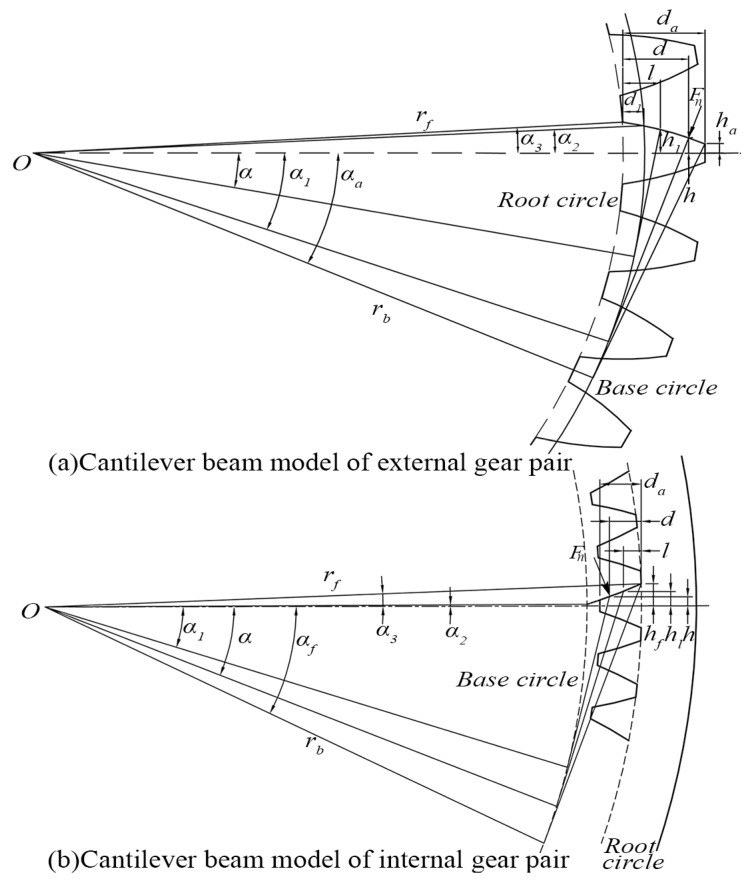
Cantilever beam model of the gear tooth.

The meshing stiffness is derived based on the energy method. The gear tooth is simplified as a cantilever beam on the root circle and the fillet is simplified as a straight-line segment, as shown in Figure 3.

In Figure 3, $F_{n}$ is the meshing force. $d_{a}$ denotes the effective calculated length of the cantilever beam model. *α* represents the half-tooth angle of the calculation point. The range from the base circle to the root circle is simplified as a straight-line segment since it is difficult to represent this range using a specific and accurate equation, which can be denoted as $d_{l}$. $k_{b}$, $k_{s}$ and $k_{a}$ express the bending stiffness, shear stiffness, and axial compression stiffness of the gear tooth, respectively. The expressions can be derived as follows:

(5) $$\frac{1}{k_{b}}=\int_{0}^{d}\frac{{\left[\cos\alpha_{1}\left(d-l\right)-\sin\alpha_{1}h\right]}^{2}}{EI_{l}}dl$$

(6) $$\frac{1}{k_{s}}=\int_{0}^{d}\frac{1.2\cos^{2}\alpha_{1}}{GA_{l}}dl$$

(7) $$\frac{1}{k_{a}}=\int_{0}^{d}\frac{\sin^{2}\alpha_{1}}{EA_{l}}dl$$

where *d* is the distance between the contact point to the root circle. *l* is the distance from the section area to the root circle. $\alpha_{1}$ means the angle between the vertical line of LOA and the tooth centerline, which is numerically equal to the meshing angle at the meshing point. *h* represents the distance from the contact point to the tooth centerline. *E* and *G* represent Young’s modulus and shear modulus, respectively. $A_{l}$ and $I_{l}$ are the section area and the area moment of inertia of the gear tooth, which can be calculated by:

(8) $$A_{l}=2h_{l}w$$

(9) $$I_{l}=\frac{2}{3}h_{l}^{3}w$$

where $h_{l}$ is the distance from the section area to the tooth centerline, and *w* is the tooth width.

According to the characteristics of involutes, for external meshing pairs, $h,h_{l},d,l$ can be obtained as:

(10) $$h=r_{b}\left[\left(\alpha_{1}+\alpha_{2}\right)\cos\alpha_{1}-\sin\alpha_{1}\right]$$

(11) $$h_{l}=\left\{\begin{array}{l} r_{b}\sin\alpha_{2}\;\;\;\;\;\;\;\;\;\;\;\;\;\;\;\;\;\;\;\;\;\;\;\;\;\;\;\;\;\;\;\;\;0\leq l\leq d_{1}\\ r_{b}\left[\left(\alpha_{2}-\alpha \right)\cos\alpha +\sin\alpha \right]\;\;\;d_{1}\leq l\leq d \end{array}\right.$$

(12) $$d=r_{b}\left[\left(\alpha_{1}+\alpha_{2}\right)\sin\alpha_{1}+\cos\alpha_{1}\right]-r_{f}\cos\alpha_{3}$$

(13) $$l=\left\{\begin{array}{l} r_{b}\sin\alpha -r_{f}\cos\alpha_{3}\;\;\;\;\;\;\;\;\;\;\;\;\;\;\;\;\;\;\;\;\;\;\;\;\;\;\;\;\;\;\;\;\;\;0\leq l\leq d_{1}\\ r_{b}\left[\cos\alpha -(\alpha_{2}-\alpha \right)\sin\alpha ]-r_{f}\cos\alpha_{3}\;\;\;d_{1}\leq l\leq d \end{array}\right.$$

(14) $$d_{1}=r_{b}\cos\alpha_{2}-r_{f}\cos\alpha_{3}$$

(15) $$\alpha_{2}=\frac{\pi }{2z}+inv\alpha_{0}$$

(16) $$\alpha_{3}=\arcsin\left(\frac{r_{b}\sin\alpha_{2}}{r_{f}}\right)$$

where $r_{b}$ and $r_{f}$ are the radius of the base circle and root circle, respectively. $d_{1}$ denotes the length from the fillet to the dedendum. $\alpha_{2}$ and $\alpha_{3}$ represent the half-tooth angle of the base circle and the root circle, respectively.

For the internal meshing pair, the equations for the corresponding parameters are as follows:

(17) $$h=r_{b}\left[\left(\alpha_{2}-\alpha_{1}\right)\cos\alpha_{1}+\sin\alpha_{1}\right]$$

(18) $$h_{l}=r_{b}\left[\left(\alpha_{2}-\alpha \right)\cos\alpha +\sin\alpha \right]$$

(19) $$d=r_{f}\cos\alpha_{3}-r_{b}\left[\cos\alpha_{1}-\left(\alpha_{2}-\alpha_{1}\right)\sin\alpha_{1}\right]$$

(20) $$l=r_{f}\cos\alpha_{3}-r_{b}\left[\cos\alpha -\left(\alpha_{2}-\alpha \right)\sin\alpha \right]$$

(21) $$\alpha_{2}=\frac{\pi }{2z}-inv\alpha_{0}$$

(22) $$\alpha_{3}=\alpha_{2}+inv\alpha_{f}$$

According to the Hertz contact theory, the Hertzian contact stiffness is shown as:

(23) $$\frac{1}{k_{h}}=\frac{4\left(1-v^{2}\right)}{\pi Ew}$$

where *v* is the Poisson’s ratio of the gear.

#### 2.3.2. Influence of the Time-Varying Contact Ratio on the Meshing Stiffness

**Figure 4:**
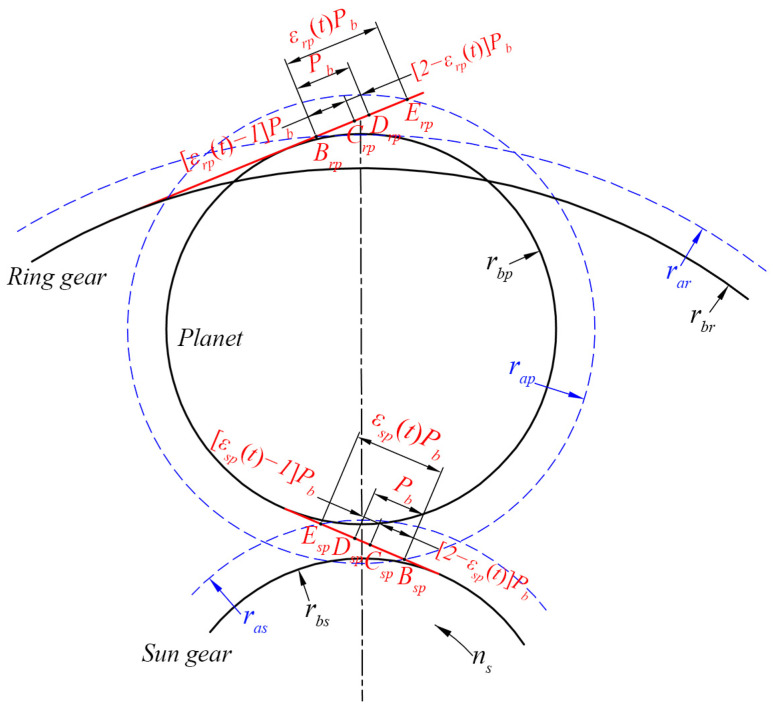
Schematic of the meshing process.

The schematic of the meshing process of the $sp_{i}$ and $rp_{i}$ meshing pairs is shown in Figure 4. $\bar{B_{g}E_{g}}\left(g=sp_{i},rp_{i}\right)$ represents the LOA. $B_{g}$ and $E_{g}$ denote the initial meshing point and terminal meshing point, respectively. The length of $\bar{B_{g}E_{g}}$ is determined by the contact ratio $\varepsilon_{g}\left(t\right)$ and base pitch $P_{b}$. During the meshing process, single- and double-tooth engagement alternates. In Figure 4, $\bar{C_{g}D_{g}}$ is the single-tooth meshing range with a length of $\left[2-\varepsilon_{g}(t)\right]p_{b}$, while $\bar{B_{g}C_{g}}$ and $\bar{D_{g}E_{g}}$ are the double-tooth meshing ranges with lengths of $\left[\varepsilon_{g}(t)-1\right]p_{b}$. According to Equation (4), the corresponding time-varying contact ratio can be calculated, and then the lengths of single- and double-tooth meshing ranges can be obtained, which are no longer constant. As a result, the period of TVMS varies.

For the single-tooth meshing range, the comprehensive meshing stiffness can be expressed as:

(24) $$k_{g}=\frac{1}{\frac{1}{k_{h}}+\sum_{i=1}^{2}\left(\frac{1}{k_{bi}}+\frac{1}{k_{s_{i}}}+\frac{1}{k_{ai}}\right)}$$

where subscripts 1 and 2 indicate the driving gear and driven gear, respectively.

Correspondingly, the duration of angular displacement in the single-tooth meshing range with the carrier eccentricity error can be expressed as:

(25) $$\theta_{g}\left(t\right)=\left[\left(n-1\right)\frac{2\pi }{z_{1}}+\left[\varepsilon_{g}\left(t\right)-1\right]\frac{2\pi }{z_{1}},n\frac{2\pi }{z_{1}}\right]\left(n=1,2,3,\ldots \right)$$

For the double-tooth meshing range, the comprehensive meshing stiffness can be expressed as:

(26) $$k_{g}=\overset{2}{\underset{j=1}{\sum }}\frac{1}{\frac{1}{k_{h,j}}+\sum_{i=1}^{2}\left(\frac{1}{k_{bi,j}}+\frac{1}{k_{si,j}}+\frac{1}{k_{ai,j}}\right)}$$

where *j* means the number of teeth pairs in the meshing.

Correspondingly, the angular displacement in the double-tooth meshing range can be expressed as:

(27) $$\theta_{g}\left(t\right)=\left[\left(n-1\right)\frac{2\pi }{z_{1}},\left(n-1\right)\frac{2\pi }{z_{1}}+\left[\varepsilon_{g}\left(t\right)-1\right]\frac{2\pi }{z_{1}}\right]\left(n=1,2,3,\ldots \right)$$

#### 2.3.3. Influence of Time-Varying Meshing Angle on the Meshing Stiffness

Due to the existence of eccentricity error in the planetary carrier, there is a periodic fluctuation in the contact angle of each meshing pair; based on Equation (3), the value of this fluctuation can be expressed as follows:

(28) $$\alpha_{vg}\left(t\right)=\alpha_{g}\left(t\right)-\alpha_{0}$$

The angle $\alpha_{1}$ is the only variable in Equations (5)–(7), which directly determines the magnitude of meshing stiffness. For the first tooth pair, the angles $\alpha_{1,1}$ and $\alpha_{2,1}$ considering the carrier eccentricity error can be expressed as follows:

(29) $$\begin{array}{l} \alpha_{1,1}=\alpha_{vg}(t)+\theta_{1}-\frac{\pi }{2z_{1}}-inv\alpha_{0}\\ +\tan\left[\cos^{-1}\frac{z_{1}\cos\alpha_{0}}{\sqrt{{\left(z_{2}+2\right)}^{2}+{\left(z_{1}+z_{2}\right)}^{2}-2\left(z_{2}+2\right)\left(z_{1}+z_{2}\right)\cos\left(\cos^{-1}\frac{z_{2}\cos\alpha_{0}}{z_{2}+2}-\alpha_{0}\right)}}\right] \end{array}$$

(30) $$\alpha_{2,1}=\alpha_{vg}(t)-\frac{z_{1}}{z_{2}}\theta_{1}-\frac{\pi }{2z_{2}}-inv\alpha_{0}+\tan\left(\cos^{-1}\frac{z_{2}\cos\alpha_{0}}{z_{2}+2}\right)$$

For the second tooth pair, the angles $\alpha_{1,2}$ and $\alpha_{2,2}$ can be expressed as follows:

(31) $$\alpha_{1,2}=\alpha_{1,1}+\frac{2\pi }{z_{1}}$$

(32) $$\alpha_{2,2}=\alpha_{2,1}-\frac{2\pi }{z_{2}}$$

where $\alpha_{1,j},\alpha_{2,j}(j=1,2)$ are the meshing angle of the driving and driven gear in the *j*-th tooth pair, respectively. $\theta_{1}$ denotes the angular displacement of the driving gear.

Combined with the analysis in Section 2.3.2, the TVMS considering the carrier eccentricity error can be calculated by substituting Equations (29)–(32) into Equations (5)–(22).

### 2.4. TVMS Model of Cracked Gear Teeth

**Figure 5:**
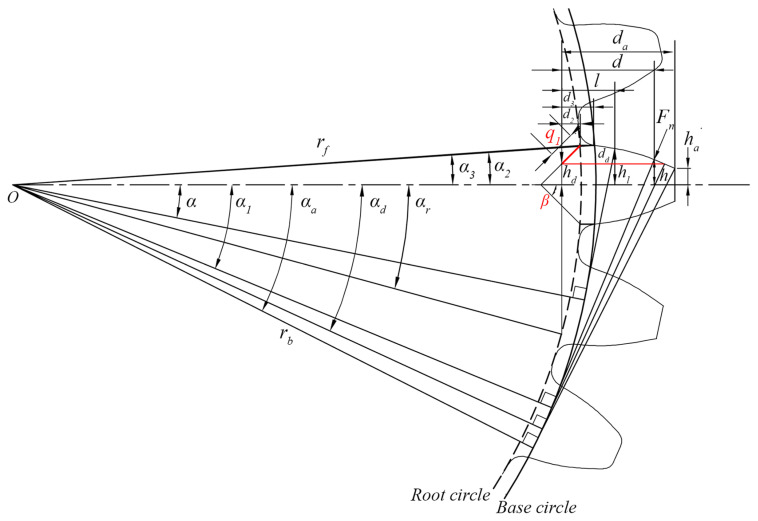
The crack propagates before the centerline of the gear tooth.

**Figure 6:**
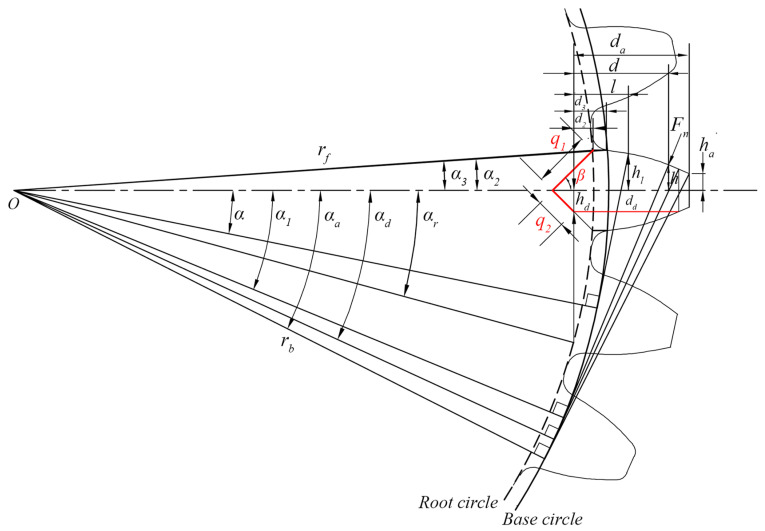
The crack propagates after the centerline of the gear tooth.

As studied in [25,26], the initial crack forms from the root of the gear tooth, and propagates along a straight line until it exceeds the tooth centerline, then continues to spread symmetrically with the tooth centerline. Figure 5 and Figure 6 show the cracked gear model before and after the centerline, respectively. $q_{1}$ is the crack length before the centerline, while $q_{2}$ is the crack length after the centerline. *β* is the angle between the crack extension line and the tooth centerline, and the value is set as 45°. $h_{d}$ represents the distance from the crack tip to the tooth centerline. The crack extension does not affect the Hertz contact stiffness and axial compression stiffness. However, it will change the section area and the area moment of inertia of the gear tooth, which will affect the bending stiffness and shear stiffness.

Situation 1: the crack is in the initial stage and propagates before the centerline.

The corresponding section area and the area moment of inertia of the gear tooth are given by:

(33) $$A_{l}=\left\{\begin{array}{ll} r_{f}\left(\sin\alpha +\sin\alpha_{3}-\frac{q_{1}}{r_{f}}\sin\beta \right)w & 0\leq l\leq d_{2}\\ r_{b}\left(2\sin\alpha_{2}-\frac{q_{1}}{r_{b}}\sin\beta \right)w\; & d_{2}<l\leq d_{3}\\ r_{b}\left[\left(\alpha_{2}-\alpha \right)\cos\alpha +\sin\alpha +\sin\alpha_{2}-\frac{q_{1}}{r_{b}}\sin\beta \right]w\; & d_{3}<l\leq d_{d}\\ 2r_{b}\left[\left(\alpha_{2}-\alpha \right)\cos\alpha +\sin\alpha \right]w & d_{d}<l\leq d \end{array}\right.$$

(34) $$I_{l}=\left\{\begin{array}{ll} \frac{1}{12}r_{f}^{3}{\left(\sin\alpha \;+\sin\alpha_{3}-\frac{q_{1}}{r_{f}}\sin\beta \right)}^{3}w & 0\leq l\leq d_{2}\\ \frac{1}{12}r_{b}^{3}{\left(2\sin\alpha_{2}-\frac{q_{1}}{r_{b}}\sin\beta \right)}^{3}w & d_{2}<l\leq d_{3}\\ \frac{1}{12}r_{b}^{3}{\left[\left(\alpha_{2}-\alpha \right)\cos\alpha +\sin\alpha +\sin\alpha_{2}-\frac{q_{1}}{r_{b}}\sin\beta \right]}^{3}w & d_{3}<l\leq d_{d}\\ \frac{2}{3}r_{b}^{3}{\left[\left(\alpha_{2}-\alpha \right)\cos\alpha +\sin\alpha \right]}^{3}w & d_{d}<l\leq d \end{array}\right.$$

where $d_{2}$ and $d_{3}$ represent the distance from the crack tip to the base circle and root circle, respectively, and can be calculated by Equations (35) and (36). $d_{d}$ represents the length of the crack tip stress boundary line, which can be calculated by Equation (40). In addition, *l* and *d* can be expressed by Equations (38) and (39), respectively.

(35) $$d_{2}=r_{f}\cos\alpha_{3}-r_{f}\cos\alpha_{r}$$

(36) $$d_{3}=r_{b}\cos\alpha_{2}-r_{f}\cos\alpha_{r}$$

(37) $$d_{d}=r_{d}\left(\alpha_{d}+\alpha_{2}\right)\sin\alpha_{d}$$

(38) $$l=\left\{\begin{array}{ll} r_{\;}\cos\alpha -r_{f}\cos\alpha_{r} & 0\leq l\leq d_{2}\\ r_{b}\cos\alpha -r_{f}\cos\alpha_{r} & d_{2}<l\leq d_{3}\\ r_{b}\left[\cos\alpha -\left(\alpha_{2}-\alpha \right)\sin\alpha \right]-r_{f}\cos\alpha_{r} & d_{3}<l\leq d \end{array}\right.$$

(39) $$d=r_{b}\left[\left(\alpha_{1}+\alpha_{2}\right)\sin\alpha_{1}+\cos\alpha_{1}\right]-r_{f}\cos\alpha_{r}$$

where $\alpha_{r}$ and $\alpha_{d}$ represent the half-tooth angle of the crack tip and the stress boundary point on the crack, respectively, and can be calculated by:

(40) $$r_{b}\left(\alpha_{d}+\alpha_{2}\right)\cos\alpha_{d}=r_{b}\left(\sin\alpha_{d}+\sin\alpha_{2}\right)-q_{1}\sin\beta$$

(41) $$r_{f}\cos\alpha_{r}=r_{f}\cos\alpha_{3}-q_{1}\cos\beta$$

Situation 2: the crack is severe and propagates after the centerline.

The corresponding section area and the area moment of inertia of the gear tooth are given by:

(42) $$A_{l}=\left\{\begin{array}{ll} r_{f}\left(\sin\alpha -\frac{q_{2}}{r_{f}}\sin\beta \right)w & 0\leq l\leq d_{2}\;\\ r_{b}\left(\sin\alpha_{2}-\frac{q_{2}}{r_{b}}\sin\beta \right)w & d_{2}<l\leq d_{3}\\ r_{b}\left[\left(\alpha_{2}-\alpha \right)\cos\alpha +\sin\alpha -\frac{q_{2}}{r_{b}}\sin\beta \right]w & d_{3}<l\leq d_{d}\\ 2r_{b}\left[\left(\alpha_{2}-\alpha \right)\cos\alpha +\sin\alpha \right]w & d_{d}<l\leq d \end{array}\right.$$

(43) $$I_{l}=\left\{\begin{array}{ll} \frac{1}{12}r_{f}^{3}{\left(\sin\alpha -\frac{q_{2}}{r_{f}}\sin\beta \right)}^{3}w & 0\leq l\leq d_{2}\\ \frac{1}{12}r_{b}^{3}{\left(\sin\alpha_{2}-\frac{q_{2}}{r_{b}}\sin\beta \right)}^{3}w & d_{2}<l\leq d_{3}\\ \frac{1}{12}r_{b}^{3}{\left[\left(\alpha_{2}-\alpha \right)\cos\alpha +\sin\alpha -\frac{q_{2}}{r_{b}}\sin\beta \right]}^{3}w & d_{3}<l\leq d_{d}\\ \frac{2}{3}r_{b}^{3}{\left[\left(\alpha_{2}-\alpha \right)\cos\alpha +\sin\alpha \right]}^{3}w & d_{d}<l\leq d \end{array}\right.$$

where $l,d_{2},d_{3},d,d_{d}$ can be expressed by Equations (35)–(39), and $\alpha_{d},\alpha_{r}$ can be calculated by:

(44) $$r_{b}\left(\alpha_{d}+\alpha_{2}\right)\cos\alpha_{d}=r_{b}\sin\alpha_{d}+q_{2}\sin\beta$$

(45) $$r_{f}\cos\alpha_{r}=r_{f}\cos\alpha_{3}-\left(q_{1}-q_{2}\right)\cos\beta$$

Substituting the above equations of $A_{l}$ and $I_{l}$ into Equations (5)–(7), the bending stiffness and shear stiffness of the cracked gear can be obtained.

The crack level is defined as:

(46) $$Crack\;level=\frac{q_{i}}{q_{\max}}\times 100\%$$

where $q_{\max}$ is the maximum length of the crack across the whole tooth.

## 3. Dynamic Modeling

**Figure 7:**
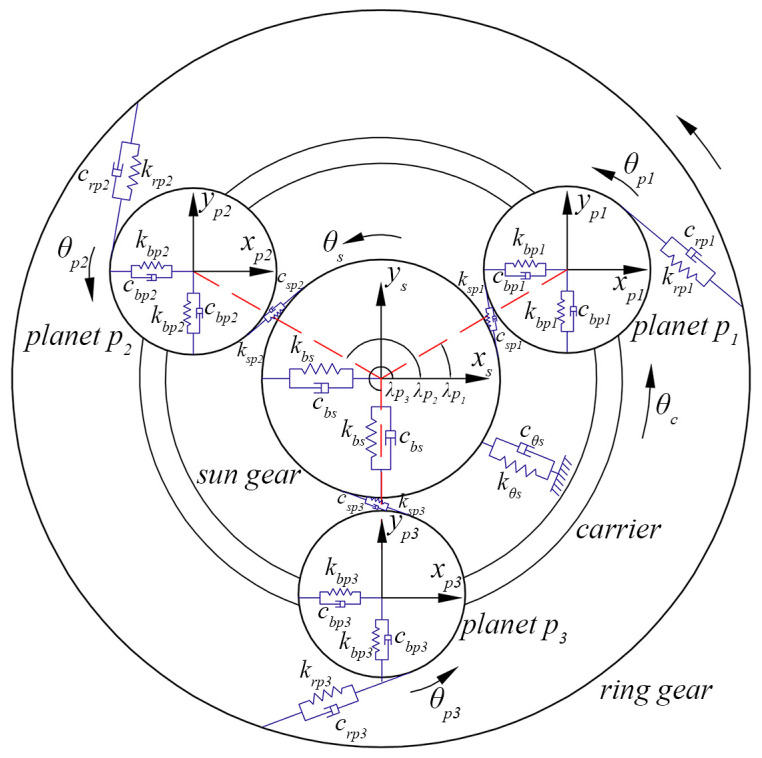
Dynamic model of a PGT.

In the above sections, the improved TVMS model considering the carrier eccentricity error and gear cracks is obtained. In this section, the relative displacement of a meshing pair considering this error is derived. On this basis, the PGT dynamic model shown in Figure 7 will be established to investigate the coupling effect of the compound fault and the planetary carrier eccentricity error. The assumptions are as follows: (1) all gears of the PGT are standard involute cylindrical spur gears; (2) the meshing relationship of each meshing pair is represented by a spring-damping structure, and the damping coefficient is linearly related to the TVMS; (3) the support of the shaft or bearing is simplified as a spring-damping structure with constant support stiffness and damping coefficient; (4) each gear component has three degrees of freedom, which includes two lateral degrees and one torsional degree of freedom denoted as x,y,θ, respectively; (5) the friction force and other gear errors are ignored in this model.

### 3.1. Relative Displacement of a Meshing Pair with the Carrier Eccentricity Error

**Figure 8:**
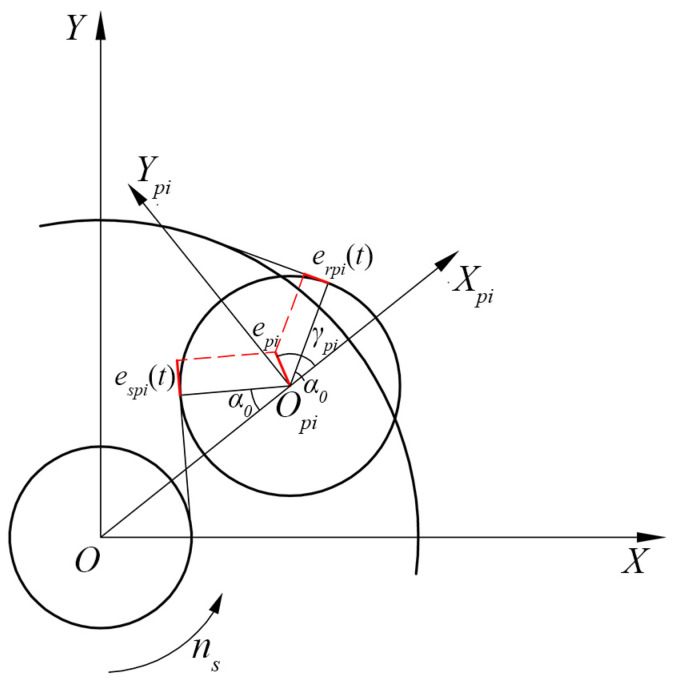
Relative displacements of meshing pairs with the carrier eccentricity error.

Based on the analysis in Section 2.2, the carrier eccentricity error can cause the planet $p_{i}$ to deviate from its theoretical meshing position, thereby affecting the LOA. Consequently, the relative displacement of the meshing pair will be directly changed since it is measured along the LOA direction. In this section, the carrier eccentricity error is incorporated into the relative displacement of a meshing pair by mapping the error on the corresponding LOA, as shown in Figure 8. In this section, *XOY* is the absolute coordinate of the system, and $X_{pi}O_{pi}Y_{pi}$ is the planet coordinate system keeping relative static with the carrier. The anticlockwise direction and the compress direction of LOA are assumed to be positive.

The planet $p_{i}$ deviation vector in $X_{pi}O_{pi}Y_{pi}$ is defined by two parameters, the magnitude $e_{pi}$ and phase angle $\gamma_{pi}$, which can be calculated as follows:

(47) $$\left\{\begin{array}{c} e_{pi}=\sqrt{{\left(x_{ei}-x_{i}\right)}^{2}+{\left(y_{ei}-y_{i}\right)}^{2}}\\ \gamma_{pi}=\tan^{-1}\left(\frac{y_{ei}-y_{i}}{x_{ei}-x_{i}}\right) \end{array}\right.$$

where $\left(x_{i},y_{i}\right)$ denotes the theoretical meshing position and $\left(x_{ei},y_{ei}\right)$ is the actual meshing position of planet $p_{i}$ in *XOY*, which can be calculated as:

(48) $$\left\{\begin{array}{l} x_{i}=a_{0}\cos\left(\omega_{c}t\right)\\ y_{i}=a_{0}\sin\left(\omega_{c}t\right) \end{array}\right.$$

(49) $$\left\{\begin{array}{l} x_{ei}=a_{pi}(t)\cos\left[\omega_{c}t-\sin^{-1}\left(\frac{e\sin\gamma }{a_{pi}(t)}\right)\right]+e\cos\gamma \\ y_{ei}=a_{pi}(t)\sin\left[\omega_{c}t-\sin^{-1}\left(\frac{e\sin\gamma }{a_{pi}(t)}\right)\right]+e\sin\gamma \end{array}\right.$$

The time-varying equivalent displacement mapping from the carrier eccentricity error to the LOA of the $sp_{i}$ meshing pair can be expressed as:

(50) $$e_{spi}\left(t\right)=-e_{pi}\sin\left(\alpha_{0}+\gamma_{pi}\right)$$

Similarly, the time-varying equivalent displacement mapping from the carrier eccentricity error to the LOA of the $rp_{i}$ meshing pair can be expressed as:

(51) $$e_{rpi}\left(t\right)=e_{pi}\sin\left(\alpha_{0}-\gamma_{pi}\right)$$

The relative meshing angle of the $sp_{i}$ and $rp_{i}$ meshing pairs can be defined respectively as:

(52) $$\varphi_{spi}=\omega_{c}t+\lambda_{pi}-\alpha$$

(53) $$\varphi_{rpi}=\omega_{c}t+\lambda_{pi}+\alpha$$

Consequently, the relative displacement of the $sp_{i}$ and $rp_{i}$ meshing pairs can be expressed respectively as:

(54) $$\delta_{spi}=-x_{s}\sin\varphi_{spi}+x_{pi}\sin\varphi_{spi}-y_{pi}\cos\varphi_{spi}+y_{s}\cos\varphi_{spi}+u_{s}+u_{pi}+e_{spi}\left(t\right)$$

(55) $$\delta_{rpi}=-x_{r}\sin\varphi_{rpi}+x_{pi}\sin\varphi_{rpi}-y_{pi}\cos\varphi_{rpi}+y_{r}\cos\varphi_{rpi}+u_{r}-u_{pi}+e_{rpi}\left(t\right)$$

The linear displacement components of the carrier relative to the planet in the *x* and *y* directions are represented as follows:

(56) $$\delta_{cp_{i}x}=x_{c}-x_{pi}-\sin\varphi_{pi}u_{c}$$

(57) $$\delta_{cp_{i}y}=y_{c}-y_{pi}-\cos\varphi_{pi}u_{c}$$

where $x_{j}$ and $y_{j}(j=c,r,s,p_{i})$ represent the displacement of component *j* in *x* and *y* directions, respectively. $u_{j}=r_{bj}\phi_{j}$ is the linear displacement of component *j* in the rotational direction, where $r_{bc}$ represents the distance between the center of planet $p_{i}$ and the carrier’s centroid. $\phi_{j}$ represents the angle at which component *j* has rotated.

### 3.2. Dynamic Equations of Motion

For $sp_{i}$ and $rp_{i}$ meshing pairs, the meshing forces always act along the LOA, which can be expressed, respectively, as:

(58) $$\left\{\begin{array}{l} F_{sp_{i}}=c_{sp_{i}}{\dot{\delta }}_{sp_{i}}+k_{sp_{i}}\delta_{sp_{i}}\\ F_{rp_{i}}=c_{rp_{i}}{\dot{\delta }}_{rp_{i}}+k_{rp_{i}}\delta_{rp_{i}} \end{array}\right.$$

where $c_{sp_{i}}$ and $c_{rp_{i}}$ represent the mesh damping coefficient of the $sp_{i}$ and $rp_{i}$ meshing pairs, and can be expressed, respectively, as:

(59) $$c_{g}=\mu_{g}k_{g}$$

(60) $$\mu_{g}=\frac{\bar{c_{g}}}{\bar{k_{g}}}$$

(61) $$\bar{c_{g}}=2\xi \sqrt{\bar{k_{g}}\bar{m_{g}}}$$

where $\mu_{g}$ is the scale constant. $\bar{c_{g}}$ denotes the average mesh damping coefficient. ξ is the mesh damping ratio, and is taken as 0.07. $\bar{k_{g}}$ and $\bar{m_{g}}$ represent the average meshing stiffness and equivalent mass, respectively.

The components of supporting forces for the central component $j\left(j=c,r,s\right)$ in three directions are respectively denoted as $F_{bjx},F_{bjy},F_{\theta j}$ and expressed as:

(62) $$\left\{\begin{array}{l} F_{bjx}=c_{bj}{\dot{x}}_{j}+k_{bj}x_{j}\\ F_{bjy}=c_{bj}{\dot{y}}_{j}+k_{bj}y_{j}\\ F_{\theta j}=c_{\theta j}{\dot{u}}_{j}+k_{\theta j}u_{j} \end{array}\right.$$

where $k_{bj}$ and $c_{bj}$ represent the radial support stiffness and damping coefficient, respectively. $k_{\theta j}$ and $c_{\theta j}$ are the torsional support stiffness and damping coefficient, respectively.

In addition, the planets are supported by the carrier, and the components of supporting forces in the *x* and *y* directions can be expressed as:

(63) $$\left\{\begin{array}{l} F_{cp_{i}x}=c_{bp_{i}}{\dot{\delta }}_{cp_{i}x}+k_{bp_{i}}\delta_{cp_{i}x}\\ F_{cp_{i}y}=c_{bp_{i}}{\dot{\delta }}_{cp_{i}y}+k_{bp_{i}}\delta_{cp_{i}y} \end{array}\right.$$

where $k_{bpi}$ and $c_{bpi}$ represent the radial support stiffness and damping coefficient of planet $p_{i}$, respectively.

Therefore, according to Newton’s second law, the dynamic equations of motion of the PGT system can be derived as follows.

The dynamic equation of the carrier is:

(64) $$\left\{\begin{array}{l} m_{c}{\ddot{x}}_{c}+F_{cp_{i}x}+\sum_{i=1}^{N}F_{bcx}=0\\ m_{c}{\ddot{y}}_{c}+F_{cp_{i}y}+\sum_{i=1}^{N}F_{bcy}=0\\ \frac{I_{c}}{r_{bc}^{2}}-\sum_{i=1}^{N}\sin\varphi_{p_{i}}F_{cp_{i}x}+\sum_{i=1}^{N}\cos\varphi_{p_{i}}F_{cp_{i}y}+F_{\theta c}=-\frac{T_{out}}{r_{bc}} \end{array}\right.$$

The dynamic equation of the ring gear is:

(65) $$\left\{\begin{array}{l} m_{r}{\ddot{x}}_{r}-\sum_{i=1}^{N}\sin\varphi_{rp_{i}}F_{rp_{i}}+F_{brx}=0\\ m_{r}{\ddot{y}}_{r}+\sum_{i=1}^{N}\cos\varphi_{rp_{i}}F_{rp_{i}}+F_{bry}=0\\ \frac{Ir}{r_{br}^{2}}{\ddot{\theta }}_{r}+\sum_{i=1}^{N}F_{rp_{i}}+F_{\theta r}=0 \end{array}\right.$$

The dynamic equation of the sun gear is:

(66) $$\left\{\begin{array}{l} m_{s}{\ddot{x}}_{s}-\sum_{i=1}^{N}\sin\varphi_{sp_{i}}F_{sp_{i}}+F_{bsx}=0\\ m_{s}{\ddot{y}}_{s}+\sum_{i=1}^{N}\cos\varphi_{sp_{i}}F_{sp_{i}}+F_{bsy}=0\\ \frac{Is}{r_{bs}^{2}}{\ddot{\theta }}_{s}+\sum_{i=1}^{N}F_{sp_{i}}+F_{\theta s}=\frac{T_{in}}{r_{bs}} \end{array}\right.$$

The dynamic equation of planet $p_{i}$ is:

(67) $$\left\{\begin{array}{l} m_{p_{i}}{\ddot{x}}_{p_{i}}+\sin\varphi_{sp_{i}}F_{sp_{i}}+\sin\varphi_{rp_{i}}F_{rp_{i}}-F_{cp_{i}x}=0\\ m_{p_{i}}{\ddot{y}}_{p_{i}}-\cos\varphi_{sp_{i}}F_{sp_{i}}-\cos\varphi_{rp_{i}}F_{rp_{i}}-F_{cp_{i}y}=0\\ \frac{I_{p_{i}}}{r_{bp_{i}}^{2}}{\ddot{\theta }}_{pi}+F_{sp_{i}}-F_{rp_{i}}=0 \end{array}\right.$$

where $m_{j}$ and $I_{j}$ represent the mass and mass moment of inertia of component *j*, respectively. $T_{in}$ and $T_{out}$ are the external torques applied on the sun gear and the carrier, respectively.

According to Equations (64)–(67), the matrix form of the dynamic model is expressed as:

(68) $$M\ddot{Q}\left(t\right)+\left(C_{m}+C_{b}\right)\dot{Q}\left(t\right)+\left(K_{m}+K_{b}\right)Q\left(t\right)=T$$

where *M* is the mass matrix. $K_{m}$ and $K_{b}$ are the meshing stiffness matrix and support stiffness matrix, respectively. $C_{m}$ and $C_{b}$ represent the mesh damping matrix and support stiffness matrix, respectively. *Q* is the displacement matrix and *T* denotes the load matrix.

## 4. Meshing Characteristic Analysis

In this study, the specific parameters of the PGT are listed in Table 1. The sun gear rotating speed is set to 1200 rpm, and the output load torque applied on the carrier is 100 N∙M.

### 4.1. Influence of the Carrier Eccentricity Error on Meshing Parameters

**Figure 9:**
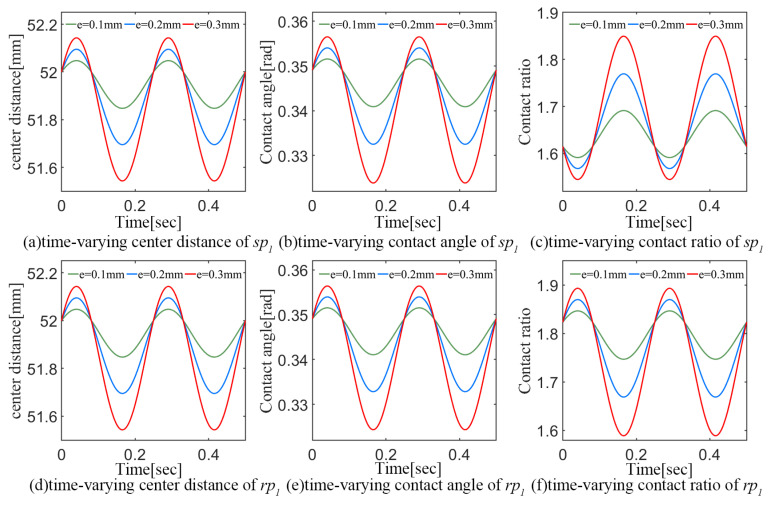
Influence of carrier eccentricity error on the meshing parameters.

Based on the calculation method in Section 2.2, the center distance, mesh angle and contact ratio of the $sp_{1}$ and $rp_{1}$ meshing pairs are shown in Figure 9 under the influence of the carrier eccentricity error with a phase angle *γ* of 45° and amplitude *e* of 0.1 mm, 0.2 mm, and 0.3 mm, respectively.

For the ideal state of the $sp_{1}$ and $rp_{1}$ meshing pairs, the center distance is 52 mm, the contact angle is 0.35, and the contact ratios are 1.61 and 1.83, respectively. However, as the planetary carrier eccentricity error acts, the meshing position of the planet will have a time-varying deviation, which is manifested in Figure 9a,d as a magnitude modulation of the center distance with a period of 0.25 s. Correspondingly, the contact angles of the $sp_{1}$ and $rp_{1}$ meshing pairs also exhibit significant fluctuations in amplitude. However, the amplitude of the contact ratio between the $sp_{1}$ and $rp_{1}$ meshing pairs shows an opposite trend of fluctuation. When the planet is affected by the error and moves away from the center of the sun gear (i.e., when the center distance increases), the contact ratio of the $sp_{1}$ meshing pair decreases while that of the $rp_{1}$ meshing pair increases.

In addition, as the amplitude of the carrier eccentricity error increases, the fluctuation range of the corresponding meshing parameters also expands, which will lead to a significant deterioration of the meshing state. The above analysis is consistent with the meshing principle of the planetary gear train, which verifies the accuracy of the proposed planetary carrier eccentricity error model.

### 4.2. Influence of the Carrier Eccentricity Error on TVMS

**Figure 10:**
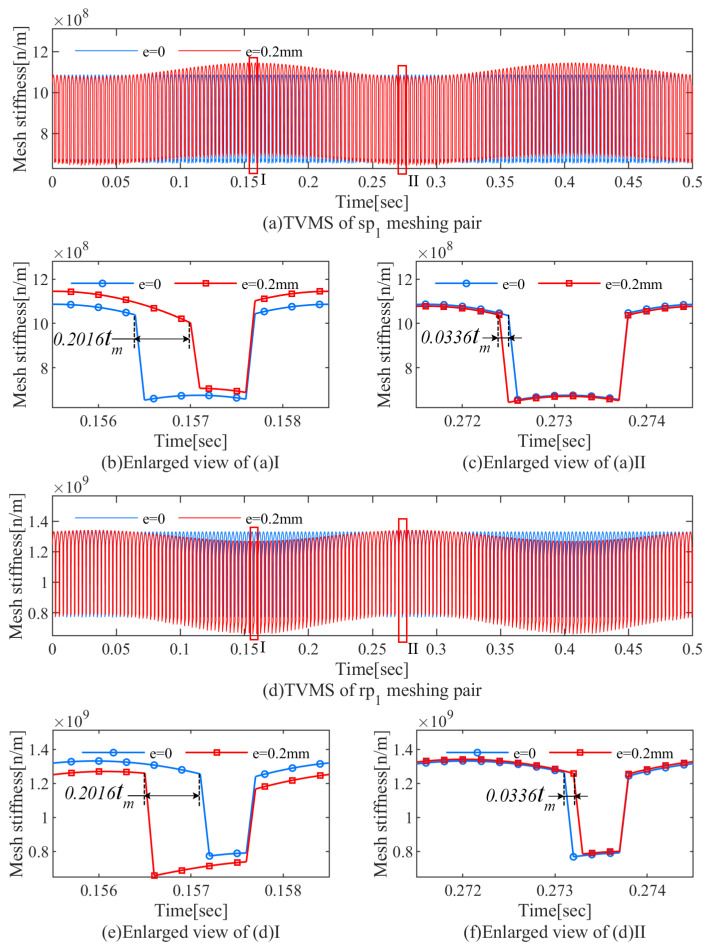
Comparisons between the TVMS without error and with e=0.2 mm.

According to the previous study, the period and magnitude of TVMS will be time-varying due to fluctuations in the contact ratio and meshing angle. Figure 10a,d shows the TVMS of the $sp_{1}$ and $rp_{1}$ meshing pairs with and without the carrier eccentricity error, respectively. Compared with the normal state, it can be observed that the carrier eccentricity error causes obvious long-term fluctuation, and its period $t_{c}=0.25\;s$ is consistent with the carrier rotation period. Furthermore, the average magnitude of the TVMS of the $sp_{1}$ meshing pair increases by 4.93%, while the average magnitude of the TVMS of the $rp_{1}$ meshing pair decreases by 5.28% compared to the normal state.

To observe the variation of the single- and double-tooth meshing ranges, several meshing periods are enlarged, as shown in Figure 10b,c,e,f, respectively.

In Figure 10b,e, when planet $p_{1}$ is engaged with the minimum center distance, the single-tooth meshing period of the $sp_{1}$ and $rp_{1}$ meshing pairs are shortened and lengthened by $0.2016t_{m}$, respectively (where $t_{m}$ denotes the meshing period). When planet $p_{1}$ is engaged with the maximum center distance due to the carrier eccentricity error, the variation of the single- and double-tooth meshing range is shown in Figure 10c,f.

### 4.3. TVMS Variation with Different Fault Combinations

The effects of the carrier eccentricity error and gear crack compound fault on the TVMS are the focus of this section. As shown in Table 2, the following six fault combinations are investigated in this section.

**Figure 11:**
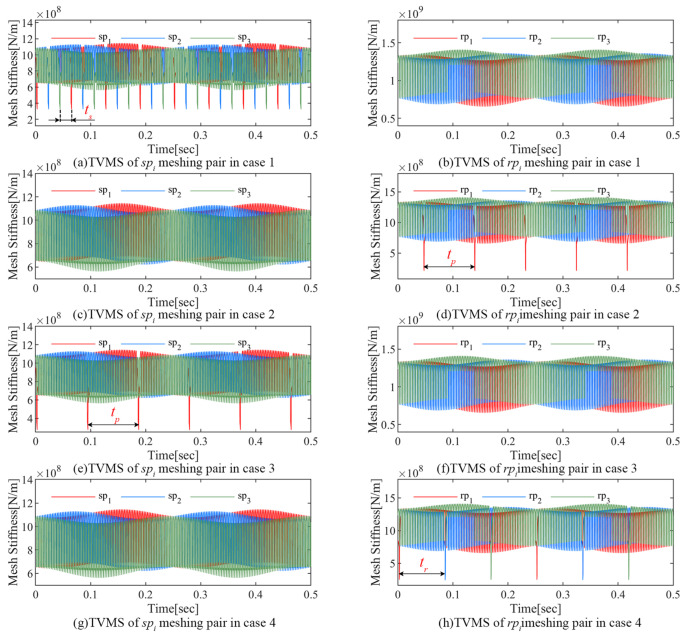
Influence of error and single gear crack on TVMS.

**Figure 12:**
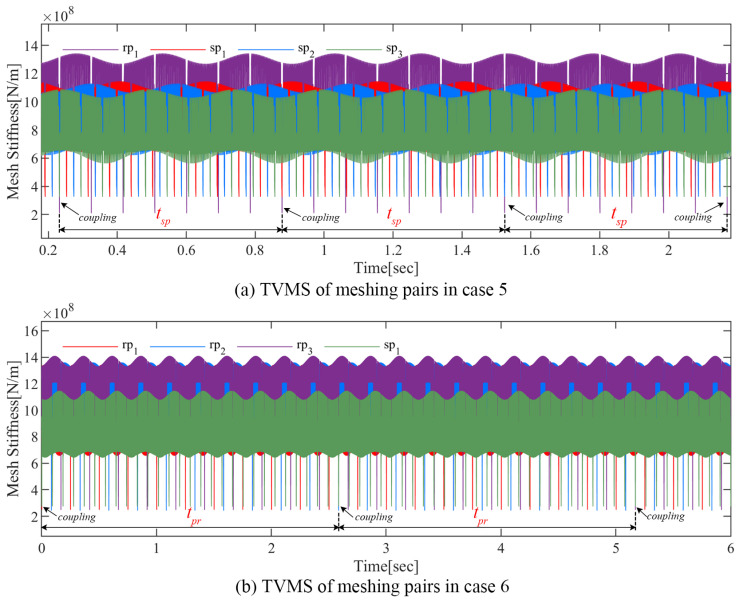
Influence of the error and compound gear cracks on TVMS.

Figure 11 and Figure 12 illustrate the TVMS in the above cases. The TVMS contains not only a short-period pulse caused by tooth engagement and a long-term modulation caused by the carrier eccentricity error, but also many complex abnormal amplitudes.

The series of abnormal pulses of period $t_{s}=0.0208\;s$ shown in Figure 11a is generated by the engagement of the cracked sun gear tooth and matched planets. As shown in Figure 11b, the sun gear crack does not affect the TVMS of the $rp_{i}$ meshing pairs.

It can be seen from Figure 11c,d that as the cracked teeth engage with the ring gear, a series of abnormal amplitudes with a period of $t_{p}=0.0922\;s$ appear in the TVMS of the $rp_{1}$ meshing pair, while the $sp_{i}$ meshing pairs are not affected.

Similarly, Figure 11e,f shows the abnormal amplitudes of the corresponding meshing pairs as the cracked planet $p_{1}$ teeth engage with the sun gear.

For the ring gear crack described in case 4, the associated TVMS is shown in Figure 11g,h. The cracked ring gear tooth engages with three planets in turn and generates abnormal amplitude of period $t_{r}=0.0833\;s$ in the TVMS of the $rp_{1}$, $rp_{2}$, and $rp_{3}$ meshing pairs in turn.

Figure 12 mainly focuses on the joint effect of the carrier eccentricity error and the compound fault on TVMS. Abnormal pulses with periods $t_{s}$ and $t_{p}$ can be observed in Figure 12a, which are caused by the sun gear crack and planet crack, respectively. Similarly, the two types of anomalous pulses in Figure 12b are generated by the engagement of the planet crack and ring gear crack, respectively. It is worth noting that when two cracked teeth are engaged simultaneously, the two types of anomalous pulses will be coupled with each other. This compound pulse, which is also periodic in nature, will lead to new fault characteristics and have a greater impact on the dynamic response of the PGT.

The TVMS evaluation of different faulty cases in this section can lay a foundation for the dynamic response analysis of the PGT in the following part.

## 5. Dynamic Simulation and Results Discussion

In this section, the Newmark-β numerical integration method is used to solve the dynamic model, and then the dynamic responses of the PGT in the health and fault states can be simulated. The dynamic characteristics are comprehensively analyzed using the velocity signal in the *y* direction, frequency spectrum, axis trajectory, and phase trajectory of the carrier. The fault case setup is the same as in Section 4.3.

### 5.1. Analysis of Characteristic Frequencies

For the convenience of subsequent analysis, the corresponding characteristic frequencies of the studied PGT appearing in the following sections are summarized in Table 3, where $f_{m}$ is the meshing frequency, $f_{c}$ is the carrier rotation frequency, and $f_{s}^{\left(r\right)}$ means the sun gear rotation frequency. The characteristic frequencies of the sun gear fault, planet fault, and ring gear fault are denoted as $f_{s}$, $f_{p}$, and $f_{r}$, respectively. The characteristic frequency of the sun gear crack and planet crack compound fault is $f_{sp}$. $f_{pr}$ denotes the characteristic frequency of the planet crack and ring gear crack compound fault. The detailed formulas for calculating the characteristic frequencies of PGT are shown in Appendix B.

### 5.2. Dynamic Characteristics of a PGT with the Carrier Eccentricity Error

**Figure 13:**
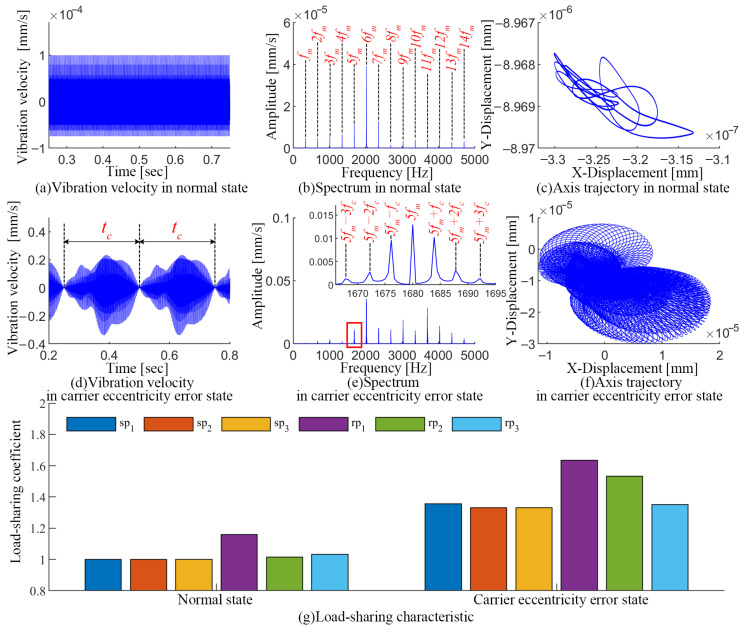
Influence of the carrier eccentricity error on the dynamic response of PGT.

In this section, the effect of the carrier eccentricity error on the dynamic characteristics is analyzed based on the time-frequency characteristics of the vibration velocity, the axial trajectory, and the load-sharing characteristics. The simulation results are shown in Figure 13.

The vibration of a PGT is mainly excited by gear meshing in a normal state, which is manifested as follows: (1) a reasonable vibration amplitude and periodic vibration waveform; (2) the spectral signals are mainly focused on the meshing frequency and its harmonics $kf_{m}$ (*k* is a positive integer), with no sideband components; (3) there is no obvious deviation in the axis trajectory; (4) the load-sharing coefficient of each meshing pair is close to the ideal value of 1.

When there is a carrier eccentricity error in a PGT, the operating condition is significantly deteriorated. A modulation effect with period $t_{c}=0.25\;s$ appears in the vibration signal, which is manifested in the spectrum as a series of sidebands located at $kf_{m}\pm mf_{c}$, where *k* and *m* are positive integers. Correspondingly, the axis of the carrier exhibits complex deviation trajectories and obvious expansion trends due to the eccentricity error. Since the carrier eccentricity error has a constant periodic effect on all the meshing pairs, the load-sharing coefficient of each meshing pair is significantly deteriorated. The maximum load-sharing coefficients for the $sp_{i}$ meshing pairs are about 1.33 to 1.35, and the load-sharing of the $rp_{i}$ meshing pairs is significantly more affected by the error, with maximum load-sharing coefficients of about 1.35 to 1.65.

### 5.3. Dynamic Responses of PGT in Different Faulty Cases

**Figure 14:**
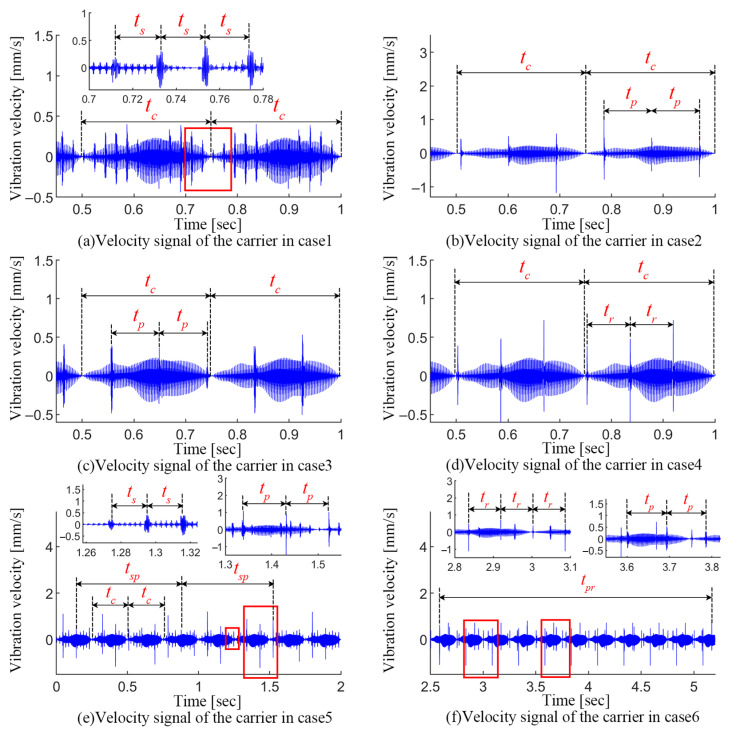
Vibration signals of PGT with different faulty cases.

**Figure 15:**
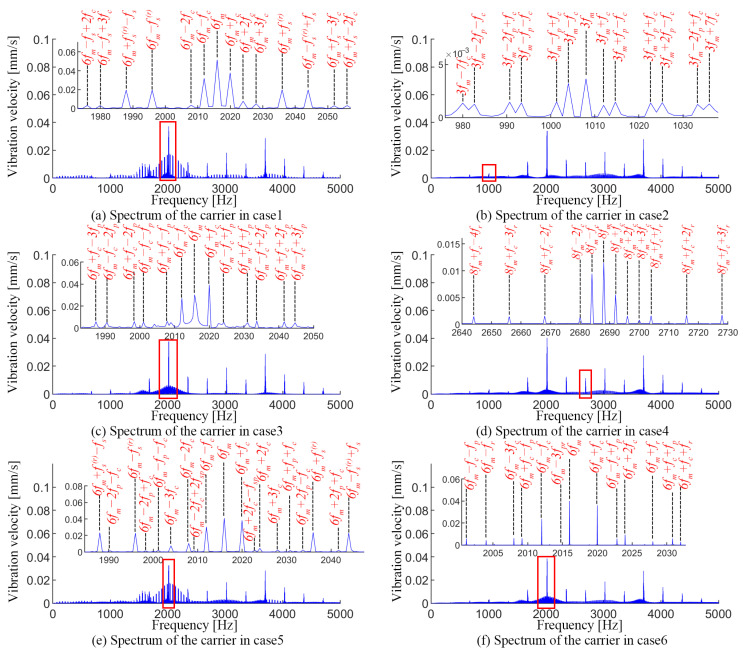
Vibration spectra of PGT with different fault settings.

This section analyzes the vibration signals shown in Figure 14 and Figure 15 for the different faulty cases. In the cases of the carrier eccentricity error and single-crack faults in the PGT, the vibration signal is predominantly excited by gear meshing, amplitude modulation effects of period $t_{c}=0.25\;s$ due to the error, and a series of periodic fault-induced impacts.

In case 1, the cracked sun gear tooth engages with the three planets sequentially, which leads to a series of impacts with period $t_{s}=0.0208\;s$ (seen in Figure 14a). A series of sidebands appear at $kf_{m}\pm mf_{c}\pm nf_{s}\pm yf_{s}^{\left(r\right)}$ in the spectrum shown in Figure 15a (where *m*, *n*, and *y* are positive integers, and not simultaneously zero), where $f_{c}$ is associated with the carrier eccentricity error. The appearance of $f_{s}$ and $f_{s}^{\left(r\right)}$ denote the sun gear crack and its accompanying modulation effect.

The dynamic responses for case 2 and case 3 are shown in Figure 14b,c and Figure 15b,c. Since the cracks on the planets in the two cases are located on the tooth faces in different directions, the cracked planet teeth engage with the sun gear and ring gear, respectively, and both generate a series of impacts with a period of $t_{p}=0.0923\;s$. Accordingly, a series of sidebands can be observed at $kf_{m}\pm mf_{c}\pm nf_{p}$ in the spectrum (where *m* and *n* are positive integers, and not simultaneously zero), which is the joint effect of the planet crack and the carrier eccentricity error.

In case 4, a series of impacts in Figure 14d with period $t_{r}=0.0833\;s$ is excited by the ring gear crack. Correspondingly, the ring gear crack interacts with the carrier eccentricity error to generate a series of sidebands in the spectrum at $kf_{m}\pm mf_{c}\pm nf_{r}$ (where *m* and *n* are positive integers, and not simultaneously zero).

For the compound faults, in addition to the impacts generated by the respective engagement of two cracked teeth, when two faults are engaged simultaneously, the impacts will be coupled and a new fault characteristic will be formed.

For the compound fault of the sun gear crack and planet crack in case 5, the velocity signal and its spectrum are shown in Figure 14e and Figure 15e, respectively. The engagement of two cracked teeth generates impacts with periods $t_{s}$ and $t_{p}$, respectively. Notably, a series of coupled impacts with a period of $t_{sp}=0.6459\;s$ are formed as two cracked gear teeth engage simultaneously with the planet and the ring gear, respectively. However, the spectrum in this case is more complicated. The sidebands mainly appear in: (1) $kf_{m}\pm mf_{c}\pm nf_{s}\pm yf_{s}^{\left(r\right)}$ (where *m*, *n*, and *y* are positive integers, and not simultaneously zero), which are mainly related to the joint effect between the carrier eccentricity error and the sun gear crack; (2) $kf_{m}\pm mf_{c}\pm nf_{p}$, which are correlated with the joint effect between the carrier eccentricity error and the planet gear crack; and (3) $kf_{m}\pm mf_{c}\pm nf_{sp}$, which indicates the superimposed impact generated by the simultaneous engagement of two cracked gear teeth.

For the compound fault of the planet crack and ring gear crack described in case 6, two types of impacts with periods $t_{p}$ and $t_{r}$ appear in the vibration velocity signal shown in Figure 14f. A portion of these impacts occurs simultaneously and results in more severe vibration amplitudes, and the period of this superimposed impacts is $t_{pr}=2.5839\;s$, which is consistent with the analytical results of TVMS in Section 4.3. Correspondingly, the sidebands in the spectrum appear mainly in $kf_{m}\pm mf_{c}\pm nf_{p}$, $kf_{m}\pm mf_{c}\pm nf_{r}$ and $kf_{m}\pm mf_{c}\pm nf_{pr}$, which indicate the joint effect of the carrier eccentricity error, planet crack, and ring gear crack.

## 6. Experimental Verification

**Figure 16:**
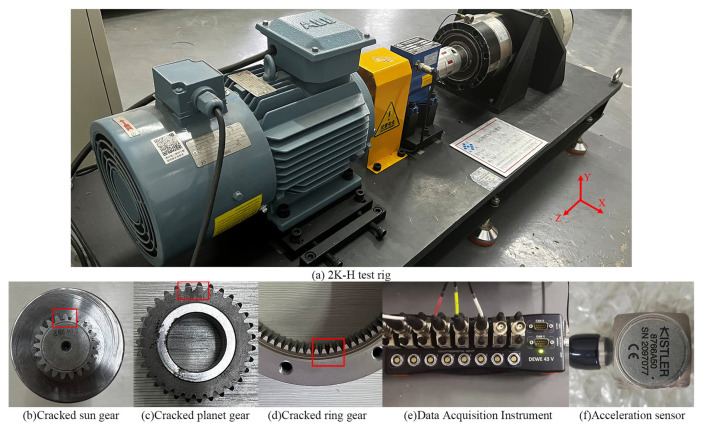
Experimental setup.

Based on the test rig shown in Figure 16a, a series of experiments are conducted to further analyze the fault characteristics. The rotation speed of the motor is 1200 rpm, and the load is 5 N∙M. Figure 16b–d shows the cracked sun gear, planet, and ring gear, respectively. The vibration signals are measured using the Dewsoft data acquisition instrument and a PCB three-axis acceleration sensor as shown in Figure 16e,f, respectively. The sampling frequency is 10,240 Hz.

**Figure 17:**
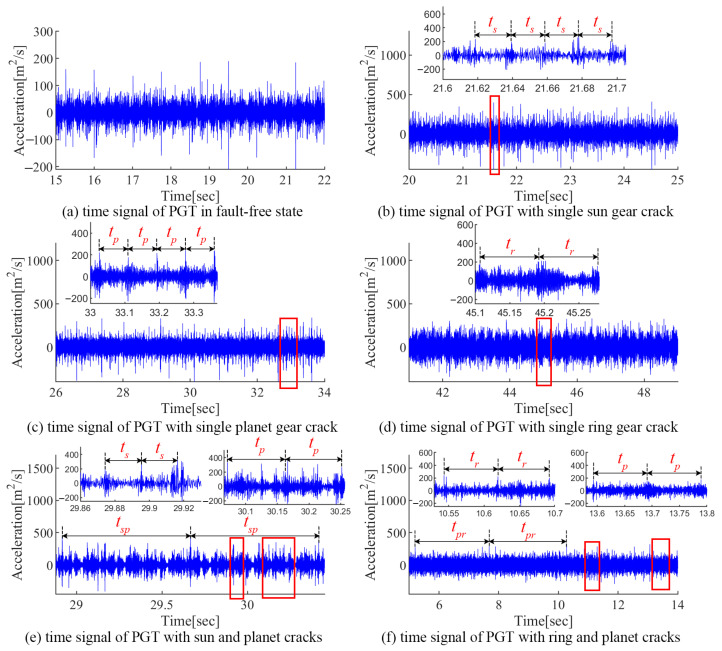
Measured acceleration signals of PGT.

**Figure 18:**
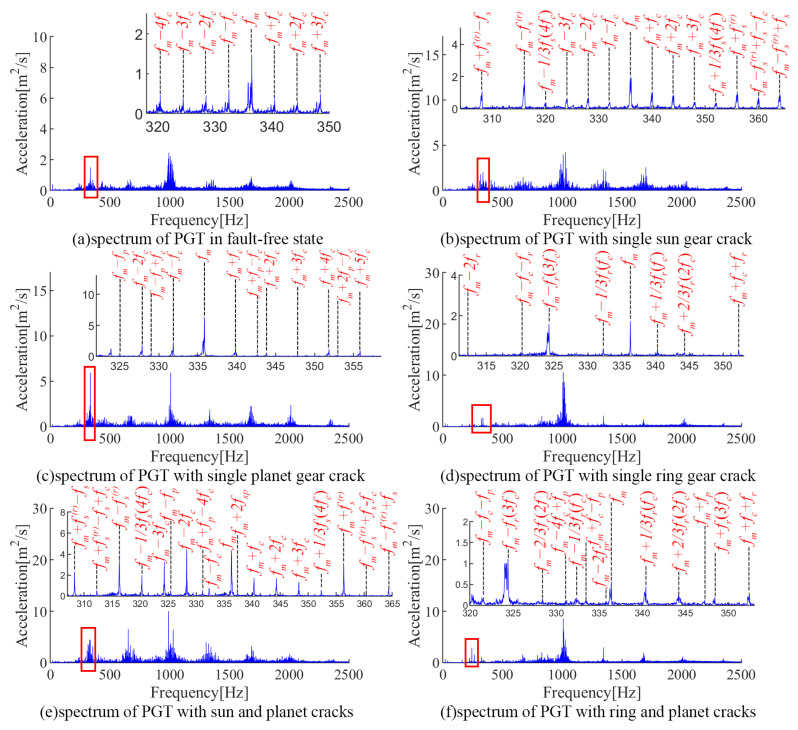
Measured spectra of PGT.

In this paper, six experiments cases are set up: a fault-free state, a sun gear crack, a planet gear crack, a ring gear crack, a compound fault of the sun gear crack and planet crack, and a compound fault of the planet crack and ring gear crack. The measured acceleration signals and the corresponding spectra are given in Figure 17 and Figure 18, respectively. Due to the influence of multi-source errors, random noise, and attenuation of the transmission path, the measured signals are more complicated and inevitably have interference components.

For the fault-free state, no obvious periodic impacts can be found in the acceleration signal (seen Figure 17a). The spectrum of the experimental signal is more complex than in the simulation due to environmental noise (seen Figure 18a). The spectrum lines located at $kf_{m}\pm mf_{c}$ (where *k* and *m* are positive integers) are obvious, which are consistent with the modulation features caused by the carrier eccentricity error.

For the sun gear crack state, fault-induced impacts with period $t_{s}$ can be observed in the acceleration signals, separately. The spectrum lines are mainly located at $kf_{m}\pm mf_{s}^{\left(r\right)}\pm nf_{s}\pm yf_{c}$, where *k*, *m*, *n*, and *y* are positive integers, and not simultaneously zero (seen Figure 18b). In addition, the inevitable manufacturing differences of the planetary gear led to the difference between the cracked gear teeth and the mesh impact of the three planetary gears, which leads to the appearance of $1/3f_{s}$, which is numerically equal to $4f_{c}$, so the relevant sidebands are $kf_{m}\pm m/3f_{s}(4mf_{c})$.

For the planet crack state, impacts with period $t_{p}$ can be observed in the acceleration signal (seen Figure 17c). Accordingly, the vibration energies are mainly located at $kf_{m}\pm mf_{p}\pm nf_{c}$ in the spectrum (seen Figure 18c).

When the ring gear is cracked, the impact signal with period $t_{r}$ can be observed in the acceleration signal in Figure 17d. Since $f_{r}$ and $3f_{c}$ are numerically equal, the sidebands in the spectrum are mainly located at $kf_{m}\pm mf_{r}(3mf_{c})\pm nf_{c}$. Similar to the sun gear crack state, the difference between three planets causes the appearance of $kf_{m}\pm m/3f_{r}(mf_{c})$.

As for the compound fault of the sun gear crack and planet crack shown in Figure 17e and Figure 18e, there are three types of impacts in the acceleration signal with periods of $t_{s}$, $t_{p}$, and $t_{sp}$, respectively. Correspondingly, the sidebands are mainly located in $kf_{m}\pm mf_{s}^{\left(r\right)}\pm nf_{s}\pm yf_{c}$, $kf_{m}\pm m/3f_{s}(4mf_{c})$, $kf_{m}\pm mf_{c}\pm nf_{p}$ and $kf_{m}\pm mf_{c}\pm nf_{sp}$, where *k*, *m*, *n*, and *y* are positive integers, and not simultaneously zero.

As the PGT contains the compound fault of the ring gear crack and planet crack, multiple types of impacts with periods $t_{p}$, $t_{r}$, and $t_{pr}$ can be observed in the acceleration signal shown in Figure 17f. Accordingly, the vibration energy is mainly located at $kf_{m}\pm mf_{c}\pm nf_{p}$, $kf_{m}\pm mf_{r}(3mf_{c})\pm nf_{c}$, $kf_{m}\pm m/3f_{r}(mf_{c})$, and $kf_{m}\pm mf_{c}\pm nf_{pr}$ in the spectrum (seen Figure 18f).

The above experimental analysis is basically consistent with the simulation results, verifying the joint effect between the carrier eccentricity error and multiple gear faults.

## 7. Conclusions

In this paper, a carrier eccentricity error model is proposed considering the time-varying center distance, LOA, meshing angle, and contact ratio. Then, an improved TVMS model considering both the carrier eccentricity error and compound gear cracks is established based on the potential energy method. On this basis, a series of dynamic simulations and experiments are conducted to investigate the faulty characteristics of the carrier eccentricity error and compound faults. The main conclusions can be summarized below.

(1) The carrier eccentricity error comprehensively changes multiple meshing parameters, which will lead to the amplitude fluctuation and variation of single- and double-tooth meshing ranges of the TVMS.
(2) The single gear crack can cause periodic TVMS reductions. Furthermore, the overlap of TVMS reductions occurs when multiple cracked teeth enter engagement simultaneously, which will cause new fault characteristics and have a greater influence on the dynamic response of the system.
(3) The amplitude modulation caused by the carrier eccentricity error can generate sidebands around meshing harmonics with an interval of rotation frequency of the carrier in the spectrum. The gear cracks induce a series of periodic impacts in the time-domain signal, and the joint effect of the carrier eccentricity error and gear cracks makes the sidebands more complex. The coupling of this effect with gear cracks generates more complex sidebands, while the coupling of different gear cracks generates a new set of impact components and causes severe degradation of the system performance.

Above all, the compound fault mechanism and the influence of carrier eccentricity error on the fault characteristics of the PGT are investigated, which can provide a reference for compound fault diagnosis in practice.

## Figures and Tables

**Table 1:** Parameters of the studied PGT.

Parameter	Sun Gear	Planet	Ring Gear	Carrier
Number of teeth	21	31	84	/
Mass (kg)	0.208	0.471	1.133	1.543
Base circle radius (mm)	19.7	29.1	78.9	/
Mass moment of inertia (kg/m^2^)	5.183 × 10^−5^	1.733 × 10^−4^	4.235 × 10^−3^	1.167
Module (mm)	2			
Tooth width (mm)	22			
Theoretical meshing angle (deg.)	20			
Theoretical contact ratio (deg.)	$\varepsilon_{sp}=1.61,\varepsilon_{rp}=1.82$			
Theoretical center distance (mm)	52			
Support stiffness (N/m)	$k_{bj}=10^{8},k_{\theta s},k_{\theta p},k_{\theta c}=0,k_{\theta r}=10^{10}$			
Young’s modulus (GPa)	206			
Poisson’s ratio	0.3			

**Table 2:** Indication of Fault Settings.

Case	Carrier Eccentricity Error	Types of Tooth Damage
1	*e* = 0.2 mm, *γ* = 45°	sun gear crack (20%)
2	*e* = 0.2 mm, *γ* = 45°	$p_{1}$ crack (40%, mesh with ring)
3	*e* = 0.2 mm, *γ* = 45°	$p_{1}$ crack (40%, mesh with sun gear)
4	*e* = 0.2 mm, *γ* = 45°	ring gear crack (20%)
5	*e* = 0.2 mm, *γ* = 45°	sun gear crack (20%) +$p_{1}$ crack (40%, mesh with ring)
6	*e* = 0.2 mm, *γ* = 45°	ring gear crack (35%) +$p_{1}$ crack (25%, mesh with sun gear)

**Table 3:** Characteristic Frequencies of the PGT.

*f_m_*/Hz	*f_c_*	$f_{s}^{\left(r\right)}$	*f_s_*	*f_p_*	*f_r_*	*f_sp_*	*f_pr_*
336	4	20	48	10.8387	12	1.5483	0.387

## Data Availability

Data are contained within the article.

## Appendix A

**Nomenclature**	**Specification**
x,y,θ	Three degrees of freedom: *x*, *y* represent translational degrees of freedom, *θ* represents torsional degree of freedom
$c,r,s,p_{i},(i=1,2,3)$	Carrier, ring gear, sun gear, and planet
N	Number of planets
$z_{j}(j=r,s,p_{i})$	Teeth number of the ring gear, sun gear, and planet
$r_{j},r_{bj},r_{fj}(j=r,s,p_{i})$	The radius of the indexing circle, base circle, and root circle of component *j*
$r_{bc}$	Distance from the planet geometric center to the carrier geometric center
$o,o_{e}$	Theoretical and actual rotation center of the carrier
e,γ	Magnitude and phase angle of the carrier eccentricity error
$r_{0},r_{pi}$	Theoretical and actual rotation radius of planet $p_{i}$
$\lambda_{pi}$	Initial position angle of planet $p_{i}$
$o_{pi}\left(t\right),o_{epi}\left(t\right)$	Theoretical and actual meshing position of planet $p_{i}$ at time *t*
$\alpha_{0}$	Theoretical meshing angle
$a_{0},a_{pi}\left(t\right)$	Theoretical and actual center distance about planet $p_{i}$ at time *t*
$\alpha_{g}\left(t\right)$	Actual meshing angle of the $sp_{i}$ or $rp_{i}$ meshing pair at time *t*
$\varepsilon_{g}\left(t\right),\left(g=sp_{i},rp_{i}\right)$	Actual contact ratio of the $sp_{i}$ or $rp_{i}$ meshing pair at time *t*
$\alpha_{\mathrm{vg}}\left(t\right)$	Variation of the actual meshing angle of the $sp_{i}$ or $rp_{i}$ meshing pair at time *t*
$z_{1},\alpha_{a1}$	Number of teeth and addendum meshing angle of the driving gear
$z_{2},\alpha_{a2}$	Number of teeth and addendum meshing angle of the driven gear
$n_{s},\omega_{c}$	Rotating speed of the sun gear and carrier
$F_{n}$	Meshing force
$d_{a}$	Effective calculated length of the cantilever beam
α	Half-tooth angle of calculation point
$d_{l}$	Range from the base circle to the root circle
$k_{b},k_{s},k_{a},k_{h},k_{g}$	Bending stiffness, shear stiffness, axial compression stiffness and Hertzian contact stiffness of gears, and comprehensive meshing stiffness
*d*	Distance from the contact point to the root circle
*l*	Distance from the section area to the root circle
$\alpha_{1}$	Angle between the vertical line of the action line and the tooth center line
*h*	Distance from the contact point to the tooth centerline
$d_{1}$	Length from the fillet to the dedendum
$A_{l},I_{l}$	Section area and area moment of inertia of tooth
*E*, *G*, *v*	Young’s modulus, shear modulus, and Poisson’s ratio
$h_{l}$	Distance from the section area to the tooth centerline
*w*	Tooth width
$\alpha_{2},\alpha_{3}$	The half-tooth angle on the base circle and root circle
$p_{b}$	Base pitch
$\theta_{g}$	Angular displacement of the $sp_{i}$ or $rp_{i}$ meshing pair
$\alpha_{1,j},\alpha_{2,j}(j=1,2)$	Meshing angle of the driving and driven gear in the *j*-th tooth pair
$\theta_{1}$	Angular displacement of the driving gear
$\alpha_{a}$	Addendum meshing angle
$q_{1},q_{2}$	Length of the crack before and after the tooth centerline
*β*	Angle between the crack extension line and the tooth centerline
$h_{d}$	Distance from the crack tip to the tooth centerline
$d_{2},d_{3}$	Distance from the end of the crack extending to the base circle and root circle
$d_{d}$	Length of the crack tip stress boundary line
$\alpha_{r},\alpha_{d}$	Half tooth angle of the crack tip and stress boundary point on the tooth crack
$q_{\max}$	Maximum length of the crack across the whole tooth
*XOY*	Absolute coordinate of the system
$X_{pi}O_{pi}Y_{pi}$	Planetary gear coordinate system keeping relative static with the carrier
$\left(x_{i},y_{i}),(x_{ei},y_{ei}\right)$	Theoretical and actual meshing positions of planet $p_{i}$ in *XOY*
$e_{pi},\gamma_{pi}$	Magnitude and phase angle of the deviation vector of planet *p_i_* in $X_{pi}O_{pi}Y_{pi}$
$e_{g}\left(t\right)$	Equivalent displacement mapping of the planet deviation vector onto the acting line of $sp_{i}$ or $rp_{i}$ meshing pair
$\varphi_{g},\delta_{g}$	Relative meshing angle and relative displacement of $sp_{i}$ or $rp_{i}$ meshing pair
$\delta_{cp_{i}x},\delta_{cp_{i}y}$	Mapping of linear displacement of planet $p_{i}$ in *x* and *y* directions
$x_{j},y_{j}(j=c,r,s,p_{i})$	Displacement of component *j* in *x* and *y* directions
$\phi_{j},u_{j},\left(j=c,r,s,p_{i}\right)$	Rotation angle of component *j* and its linear displacement in the rotational direction
$F_{g}$	Meshing force of the $sp_{i}$ or $rp_{i}$ meshing pair
$c_{g},\mu_{g}$	Mesh damping coefficient and scale constant of the $sp_{i}$ or $rp_{i}$ meshing pair
$\bar{c_{g}}\bar{k_{g}},\bar{m_{g}}$	Average mesh damping coefficient, average meshing stiffness, and equivalent mass of the $sp_{i}$ or $rp_{i}$ meshing pair
ξ	Mesh damping ratio
$F_{bjx},F_{bjy},F_{\theta j},\left(j=c,r,s\right)$	Supporting force of component *j* in *x*, *y*, and θ directions
$F_{cp_{i}x},F_{cp_{i}y}$	Component of the supporting force on planet $p_{i}$ in *x* and *y* directions
$k_{bj},c_{bj}\left(j=c,r,s\right)$	Radial support stiffness and support damping coefficient of component *j*
$k_{\theta j},c_{\theta j}\left(j=c,r,s\right)$	Torsional support stiffness and support damping coefficient of component *j*
$k_{bpi},c_{bpi}$	Radial support stiffness and support damping coefficient of planet $p_{i}$
$m_{j},I_{j}\left(j=c,r,s,p_{i}\right)$	Mass and mass moment of inertia of component *j*
$T_{in},T_{out}$	Input Torque and Output Torque
$M,C_{m},C_{b},K_{m},K_{b},Q,T$	Mass matrix, mesh damping matrix, support damping matrix, meshing stiffness matrix, support stiffness matrix, displacement matrix, and load matrix
$f_{m},t_{m}$	Meshing frequency and meshing period
$f_{c},t_{c}$	Rotation frequency of the carrier and its corresponding period
$f_{p},t_{p}$	Characteristic frequency of the planet local fault and corresponding period
$f_{r},t_{r}$	Characteristic frequency of the ring gear local fault and corresponding period
$f_{s}^{\left(r\right)}$	Rotation frequency of the sun gear
$f_{s},t_{s}$	Characteristic frequency of the sun gear local fault and corresponding period
$f_{sp},t_{sp}$	Characteristic frequency and corresponding period of the sun gear and the planet gear compound fault
$f_{pr},t_{pr}$	Characteristic frequency and corresponding period of the planet and ring gear compound fault

## Appendix B

For the ideal PGT without error, the local fault characteristic frequencies of the sun gear, planet, and ring gear are denoted as $f_{s}$, $f_{p}$ and $f_{r}$, separately. The expressions are deduced as [27]:

(A1) $$f_{s}=N\frac{f_{m}}{z_{s}}$$

(A2) $$f_{p}=\frac{f_{m}}{z_{p}}$$

(A3) $$f_{r}=N\frac{f_{m}}{z_{r}}$$

where *N* is the number of planets. $f_{m}$ is the meshing frequency of the PGT, and it is calculated as:

(A4) $$f_{r}=N\frac{f_{m}}{z_{r}}$$

where $f_{s}^{\left(r\right)}$ means the rotation frequency of sun gear and $f_{s}^{\left(r\right)}=\frac{n_{s}}{60}$.

As for the sun gear and planet compound faults, when the two cracked teeth are engaged simultaneously, the corresponding impacts will be superimposed with the characteristic frequency of [28]:

(A5) $$\left\{\begin{array}{l} f_{sp}=N\frac{f_{m}}{N_{2}z_{P}}\\ N_{2}=\frac{z_{s}}{m_{2}} \end{array}\right.$$

where $m_{2}$ is the maximum common divisor of the number of sun gear teeth and planet teeth.

Similarly, the characteristic frequency of planet and ring gear compound fault is shown as:

(A6) $$\left\{\begin{array}{l} f_{pr}=N\frac{f_{m}}{N_{3}z_{P}}\\ N_{3}=\frac{z_{r}}{m_{3}} \end{array}\right.$$

where $m_{3}$ is the maximum common divisor of the number of ring gear teeth and planet teeth.
